# Gelatin and Bioactive Glass Composites for Tissue Engineering: A Review

**DOI:** 10.3390/jfb14010023

**Published:** 2022-12-31

**Authors:** Maria E. V. Barreto, Rebeca P. Medeiros, Adam Shearer, Marcus V. L. Fook, Maziar Montazerian, John C. Mauro

**Affiliations:** 1 Northeastern Laboratory for Evaluation and Development of Biomaterials (CERTBIO), Department of Materials Engineering, Federal University of Campina Grande, Campina Grande 58429-900, PB, Brazil; 2 Department of Materials Science and Engineering, The Pennsylvania State University, State College, PA 16802, USA

**Keywords:** bioactive glass, gelatin, tissue engineering, bone, composite

## Abstract

Nano-/micron-sized bioactive glass (BG) particles are attractive candidates for both soft and hard tissue engineering. They can chemically bond to the host tissues, enhance new tissue formation, activate cell proliferation, stimulate the genetic expression of proteins, and trigger unique anti-bacterial, anti-inflammatory, and anti-cancer functionalities. Recently, composites based on biopolymers and BG particles have been developed with various state-of-the-art techniques for tissue engineering. Gelatin, a semi-synthetic biopolymer, has attracted the attention of researchers because it is derived from the most abundant protein in the body, viz., collagen. It is a polymer that can be dissolved in water and processed to acquire different configurations, such as hydrogels, fibers, films, and scaffolds. Searching “bioactive glass gelatin” in the tile on Scopus renders 80 highly relevant articles published in the last ~10 years, which signifies the importance of such composites. First, this review addresses the basic concepts of soft and hard tissue engineering, including the healing mechanisms and limitations ahead. Then, current knowledge on gelatin/BG composites including composition, processing and properties is summarized and discussed both for soft and hard tissue applications. This review explores physical, chemical and mechanical features and ion-release effects of such composites concerning osteogenic and angiogenic responses in vivo and in vitro. Additionally, recent developments of BG/gelatin composites using 3D/4D printing for tissue engineering are presented. Finally, the perspectives and current challenges in developing desirable composites for the regeneration of different tissues are outlined.

## 1. Introduction

**Figure 1:**
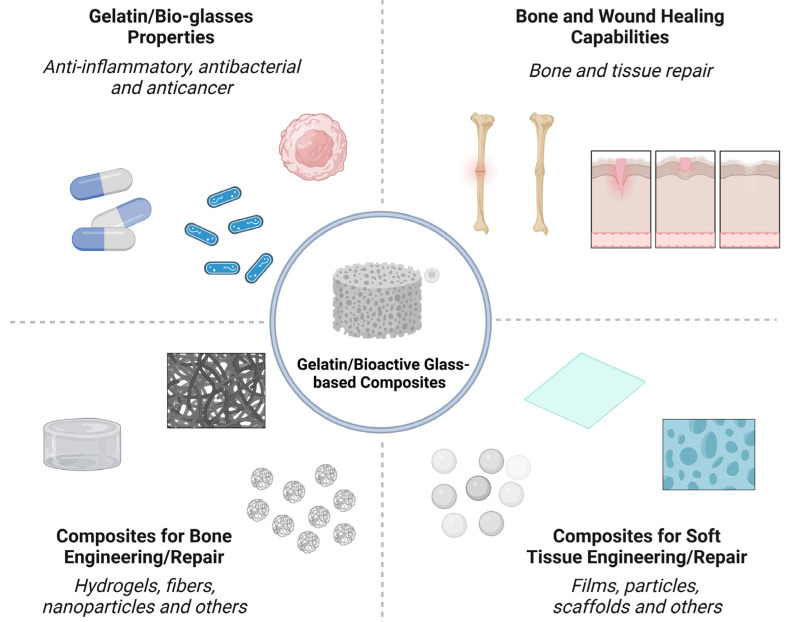
Properties, applications, and processing of gelatin/BG composites. Created by using BioRender.com.

Composites based on gelatin and bioactive glass (BG) with different morphologies and compositions have been designed to assist in the treatment of tissue injuries, aiming at the aesthetic and functional recovery of damaged limbs [1,2]. In general, these materials must be biocompatible and/or biodegradable, have mechanical strength comparable to that of the host tissue, and allow cellular activity at the implant site [3]. In addition, it is desirable that gelatin/BG composites overcome the clinical and socioeconomic limitations associated with the use of conventional applications [4]. The properties, applications, and processing of gelatin/BG composites are summarized in Figure 1 and are discussed in detail in this review.

An intrinsic characteristic of soft and hard tissues is the ability to induce regeneration mechanisms [5,6]. In bone, osteoblastic cells initiate the process from the secretion of collagen and, subsequently, the crystallization of hydroxyapatite [7]. Skin, which is the largest organ in the body, induces wound repair through the recruitment of cells that perform angiogenesis and form granulation issue, culminating in wound re-epithelialization [8].

However, the natural mechanisms of tissue recovery are restricted by the type, extent, and/or depth of the lesion, as well as by the interference of microorganisms that cause infections [9,10]. In the first case, the severity of the injury would require extensive time for tissue remodeling to occur only by induction of the organism. This could lead to residual defects, such as the formation of fibrous tissue where tissues should grow [11].

In the second hypothesis, the invasion of infectious agents during or after the implantation would impair the patient’s recovery and increase the costs associated with hospitalization and clinical procedures [12]. In the United States, annual wound repair costs exceed $50 billion [13]. Worldwide, the impacts reach enormous proportions. These numbers reinforce the demand for new products and therapeutic approaches. Added to this is the evident expansion of the biomaterials market, estimated at $106.5 billion in a 2019 evaluation [14].

The development of composites and hybrids based on gelatin and bio-glass to solve tissue engineering issues has been reported since the early 2000s [15,16,17,18]. For bone tissue, there is evidence that these materials promote more effective mineralization compared to other types of treatment [19,20]. The main justification reported for this is the chemical similarity between the composite and the organic and inorganic phases in the bone structure; thus making possible a greater reactivity with the host tissue when the composite is implanted [21]. In addition, the mechanical performance achieved with the union of the polymer with the ceramic approximates the degradation time of the material to the speed of bone formation [22].

Regarding soft tissue applications, the gelatin/BG combination has accelerated wound closure, stimulating increased angiogenesis and granulation [23]. This is due to the presence of bioactive ions capable of reducing the inflammatory response and stimulating the secretion of proteins and growth factors [24]. For example, the presence of Si promotes an upregulation in the expression of vascular endothelial growth factor (VEGF), and the difference between the results achieved only with the polymeric matrix after the addition of BG is evident [25]. In this scenario, there are many possibilities for the application of products based on gelatin and BG including: treatment of subcutaneous and cutaneous lesions [23,26], chronic wounds [27], nerve regeneration [28], muscles [29], cartilage [30] and others.

Although both gelatin and bioactive glasses have been studied extensively for their impact on both hard and soft tissue repair, many limitations remain to be addressed. Gelatin requires crosslinking of its chains to obtain the required stability in physiological environments [31,32]. For example, the thermostability of gelatin without crosslinking can change the structure from a solid to a gel which can lead to adverse effects when implanted in vivo [33,34]. The field of bioactive glass has received an overwhelming amount of attention, with hundreds of publications being published per year. All aspects including chemistry, processing, and application, have been studied, but such a broad field has left many gaps to be explored. Compositional design requires optimization depending on the desired application. For example, SiO_2_-based compositions are less suited for soft tissue regeneration than B_2_O_3_ or P_2_O_5_ [35]. Processing methods such as sol-gel synthesis have yet to be commercialized and less than 26 BG-based medical devices have been approved for clinical use [36]. 

From this perspective, the relevance of composites based on gelatin and bioactive glass applied to tissue repair requires a more comprehensive and in-depth discussion, which is not yet available in the literature. For this reason, the present work aims to provide a broader view of the development of these composites and their use in the treatment of soft and hard tissue injuries. Section 2 and Section 3 deal with regenerative biological phenomena and a brief history of existing treatment methods. Definitions and properties of gelatin and bioactive glass are described in Section 4 and Section 5, respectively. In Section 6, the research involving composites and gelatin/BG hybrids is reviewed and discussed. Finally, challenges and future perspectives on the topic are suggested (Section 7 and Section 8).

## 2. Bone Engineering

Bone tissue is one of the largest systems present in living organisms [37]. It differs from other tissues in that it is in a constant process of reconstruction, as some parts of the bone are absorbed, others are excreted and/or remodeled as a result of the dynamics of osteoblastic, osteolytic, and osteoclastic cells [7,11,37].

Bone is a natural composite, with about 70% of its composition consisting of inorganic phases based on calcium and phosphate salts. The other fraction is organic, predominately composed of type I collagen, but proteins such as proteoglycans and glycoproteins are also present. The tissue morphology is also heterogenous as it consists of some compact/dense regions (cortical bone) and other porous/spongy (trabecular bone) regions [1,38,39].

### 2.1. Bone Healing Mechanisms

When bone is damaged, whether as a result of bone loss, fractures, disease, or any other type of injury, phenomena such as hemorrhage, matrix destruction and cell death occur. From this, the regeneration process can be summarized in three continuous and simultaneous phases: inflammation, regeneration, and remodeling [5,11].

**Figure 2:**
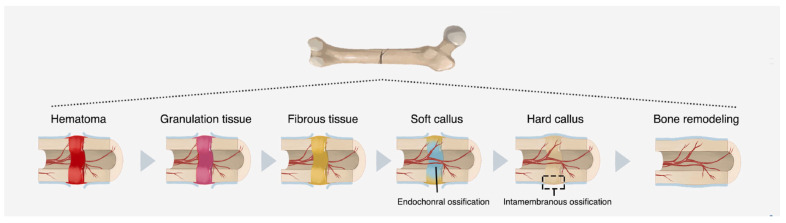
Natural process of bone repair in the fracture zone. Adapted from Zhu et al. [7].

Initially, macrophages eliminate cellular and tissue debris. Then, new osteoprogenitor cells begin to proliferate, forming connective tissue, “glue”, between the ends of the injured region [7]. Gradually, a “bone callus” is formed at the site (Figure 2), which is replaced by a secondary structure similar in shape to the one that previously existed [37].

The first phase of bone formation itself occurs when osteoblasts secrete collagen molecules and proteoglycans [7]. It is assumed that after these steps, salt deposition begins, culminating in the final product known as hydroxyapatite, with the chemical formula Ca_10_(PO_4_)_6_(OH)_2_ [39,40].

The subsequent stage is the polymerization of the excreted monomers, resulting in an osteoid, which consists of a non-mineralized matrix whose texture is similar to cartilage. Gradually, the calcium and phosphate particles deposited on the collagen matrix multiply and are distributed throughout the tissue, converting into hydroxyapatite crystals over the course of a few days or weeks. This structural characteristic confers bone tissue’s high tenacity and compressive strength [37,39,41].

The regeneration processes described above are expected to occur without the intervention of fibrous tissue and undesirable microorganisms such as bacteria. This is an important issue to be considered, given that the injuries caused to hard tissue also directly impact the socioeconomic system due to the costs of hospitalization, clinical procedures, surgeries and work disability in some cases [42].

### 2.2. Orthopedic Clinical Challenges

For hundreds of years, prosthetic implants utilized metals and their alloys with a primary emphasis on titanium, cobalt-chromium, and stainless steel. These metals had good mechanical performance but were subject to corrosion and lacked osteointegration [10,14,43,44]. From the end of the 20th century, with the creation of the tissue engineering concept, studies were directed towards the search for materials that exhibit chemical similarity with the tissue, maintain the mechanical stability of the host and lead the tissue regeneration process [43,45]. However, orthopedic problems still represent an emerging and global issue. In the 2019 World Health Organization report, injuries caused by trauma occupy the second position in the ranking of the main causes of death in the world [46]. 

Critical-sized bone defect healing represents one of the most significant unmet obstacles in bone regeneration. Originally, bone grafting was used to repair defects caused by tumors, traumatic fractures, and other types of injuries [41]. However, the technique has limitations associated with prohibitive costs and potential damage to health, resulting from infection, inflammation, or immunological rejection at the implant site [5,47].

Biomaterials are used to repair these defects and restore structure and function, often by acting as a substitute for the missing bone. The optimal characteristics for such biomaterials may differ significantly depending on the location of the bone defect and the kind of bone loss (cortical versus cancellous). If a soft biomaterial (e.g., gelatin/BGs composites) is used to fill the cortical lesion instead, a stable plate fixation is necessary to provide mechanical stability. In such a circumstance, the patient will need to be able to move around freely, which requires a rapid change of the softer biomaterial into cortical bone. In most cases, implant loosening or fatigue failure should not occur until after bone growth and consolidation have occurred. If this race is lost, incomplete osteosynthesis leads to nonunion and implant failure [48,49,50]. 

Bone loss or resection due to a tumor or infection can also result in critical-sized defects. Bone replacement is an integral aspect of treatment in these scenarios. It would be beneficial if a biomaterial could deliver substances that cure the underlying disease that causes bone loss. This functionalization of biomaterials may become one of the most important progresses in biomaterials research. Treatment for bone abnormalities following infection typically entails two or more phases of revision surgery, with antibiotic-loaded bone cement spacers used between procedures. In this case, a vascularized fibular graft is used to bypass the donor site morbidity of the autologous bone graft by using a biomaterial with bone regeneration capabilities for large defects and the elution of antibiotics [51,52,53].

Another problem is bone abnormalities in seniors because of low-impact fractures. Significant deformities sometimes result from several fractures in these people, with the underlying cause often being an osteopenic bone weakness. A commuted fracture most often occurs in the proximal femur, proximal humerus, or vertebral body. Limited bone quality in the remaining bone makes rigid fracture fixation by standard instrumentation difficult. Bone grafting, either autologous or allogeneic, is frequently used to repair these types of abnormalities, which can lead to arthroplasty in the future. Methods of enhancing bone regeneration are desperately needed considering the aging of the population and the rise in late-life activity. Given this, it is easy to appreciate the pressing need for novel therapies that give surgeons the tools they need to facilitate rapid and reliable bone regeneration in their patients [54,55,56].

## 3. Soft Tissues Engineering

Soft tissues are present in all organs that make up the body, being distinguished into four types: epithelial, connective, muscular and nervous. The epithelial tissue (or epithelium) lines the surfaces and body cavities and has the function of secreting substances. Connective tissue is located below the others, acting to support and sustain them. Muscle tissue, in turn, is responsible for body movements induced by cells capable of contracting. Nervous tissue establishes the connection between external and internal stimuli to the organism, enabling the performance of activities with different levels of complexity [37,40]. In this section, skin lesions, which predominantly consider the epithelial and connective tissues, will be discussed in greater depth. 

The skin is considered the largest organ in the body. It plays an immunological role, as it acts as a mechanical, physical and chemical barrier, protecting internal structures against infections and injuries of different nature, such as cuts, traumas, burns and ulcers [57]. In addition to functioning as an “envelope” for the body, the skin regulates moisture loss and changes in body temperature while also acting as natural mechanism to promote the reconstitution of its structure when damaged, which makes up the wound healing cascade [6,58].

The skin’s immune mechanism can be subdivided into two parts that are connected to each other and synchronized with the body’s immune system as a whole: the epidermal region and the dermal region. Both generate a favorable environment for the performance of immune cells, but also coexist cells responsible for continuous tissue maintenance and regeneration. Fibroblasts stand out as a predominant lineage in connective tissues in general, whose functions include locomotion capacity, collagen fiber production and extracellular matrix (ECM) renewal [59].

### 3.1. Wound Healing Mechanisms

**Figure 3:**
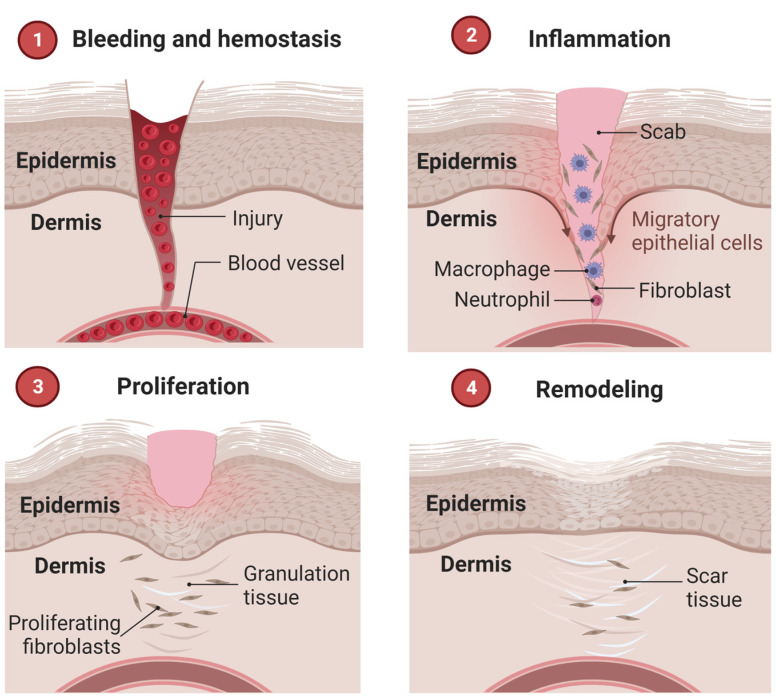
Stages of the wound healing cascade. Created by using BioRender.com.

The wound healing process occurs in well-defined phases, involving different cell types and metabolisms. Three overlapping steps are known: inflammation, proliferation, and remodeling [8,60]. Some classifications consider separately a hemostasis stage, totaling four, briefly described below and illustrated in Figure 3. The initial stage precedes inflammation and results in bleeding interruption from clot formation (hemostasis). In this scenario, activated platelets secrete cytokines that attract inflammatory cells and other populations to the wound site [58].

The inflammatory phase occurs during the initial stages of recovery after an injury, protecting it from pathogens. It is characterized by the secretion of growth factors from inflammatory cells, which stimulate the proliferation of vascular endothelial cells and fibroblasts. The latter produces type III collagen that replaces the fibrin matrix. Other cells are recruited to the site of injury at the same time, including neutrophils, monocytes, mast cells, and other non-inflammatory categories that actively contribute to the healing flow and protection against bacteria and antigens [61,62].

From cell proliferation, which consists of the second stage, angiogenesis and granulation tissue formation begin, followed by wound re-epithelialization. When there is a deficiency in the speed of cell proliferation and, consequently, in the deposition of collagen, the healing process exceeds the expected period. According to this criterion, wounds are classified as acute when recovered between 8 and 12 weeks, and chronic when healing is delayed or does not occur [13,63]. 

Finally, remodeling and/or maturation occurs, which can last up to two years after the appearance of the lesion. It is characterized by the gradual replacement of type III collagen by type I collagen, which generates a more rigid structure at the wound site and forms scar tissue [64].

### 3.2. Therapeutic Approaches in Wound Repair: A Brief Introduction

**Figure 4:**
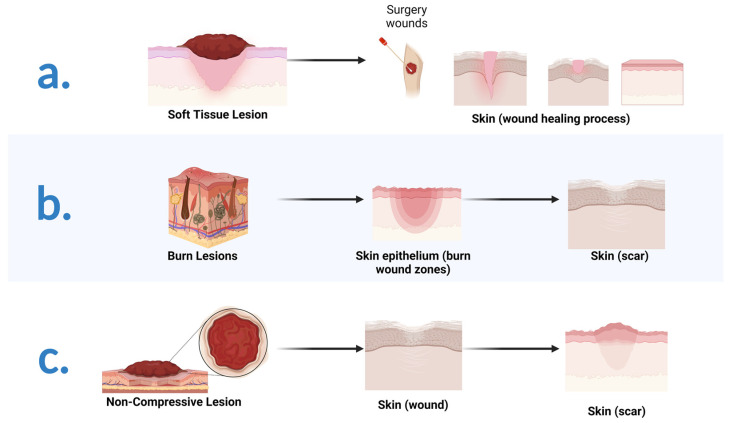
Typical injuries caused to soft tissue and healing processes: (**a**) Lesions caused by traumas, diseases, and/or accidents; (**b**) Lesions caused by burns; (**c**) Non-compressive lesions caused by sharp objects and/or firearms. Created by using BioRender.com.

To repair injuries caused to soft tissues (Figure 4a) as a result of trauma, diseases and/or accidents, one of the most used practices over time is grafting, as for hard tissue. For wound care, costs exceed $50 billion annually to serve more than 5.7 million people in the United States alone [13]. When the wound does not heal on its own, standard therapy includes debridement and skin grafting once the granulation tissue has formed [65].

However, autologous grafts can trigger complications such as infections in the postoperative phase, immunological rejection, absorption, and loss of volume. In addition, this technique is associated with a decrease in mechanical resistance, which can lead to graft failure and generate a severe scar contracture. For this reason, the scientific community in the field of tissue engineering has been dedicated to the development of systems capable of regenerating and restoring the functionality of these tissues, overcoming the limitations of practices already in use [4]. 

Until the 20th century, the treatment of burn wounds (Figure 4b) had many limitations, commonly resulting in the patient’s death due to poor wound care management. Pharmacotherapy strategies have advanced, but challenges in treating soft tissue injuries remain. As already stated, infection is a predominant issue, whether endogenous or exogenous. The multiplication of microorganisms, primarily facilitated by overly moist wound environments or delays in healing, prolongs the hospitalization period driving costs higher for the healthcare system [9,58].

A particular problem is deep wounds generated by trauma that cause uncontrolled bleeding. Currently, hemorrhage is the cause of more than 30% of deaths from trauma worldwide due to the difficulty of providing the patient with immediate intervention and prior to hospital care [24]. 

Especially in cases of non-compressible injuries (Figure 4c), such as those caused by sharp objects and/or firearms, conventional dressing methods and direct pressure are inefficient, which reinforces the demand for hemostatic agents capable of stopping acute bleeding and promoting healing, minimizing the risk of bacterial colonization at the wound site [66].

Additionally, the type of injury is also a limiting factor to allow healing by conventional methods. In the case of extensive and/or deep wounds, there is a shortage of healthy tissue available for autogenous grafting; that is, those whose source of extraction is the patient themself. In the cases of large tissue loss, the wound does not heal by primary intention, measured by approximation by edges of the suture [63]. These instances require intensive care to promote secondary intention where granulation grows at the edge of the open wound. To meet this demand, new approaches have been explored including implants, dressings, artificial organs and living tissue, which are created by growing cells in scaffolds before insertion into the body [13,61]. Among the candidate materials for repairing soft and hard tissue injuries, composites based on gelatin and bioactive glass have been extensively explored. Their potential is thoroughly discussed after briefly addressing the gelatin and BGs characteristics.

## 4. Gelatin

**Figure 5:**
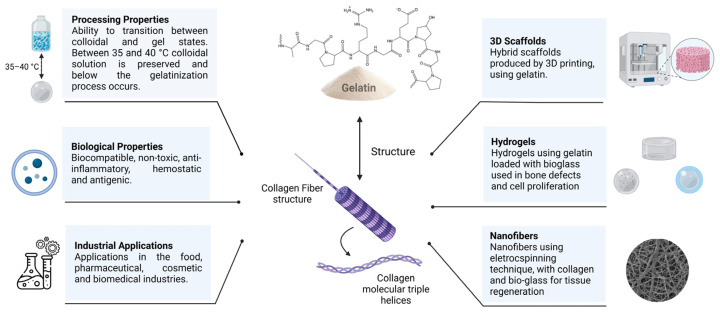
Structure, properties and applications of gelatin. Created by using BioRender.com.

Gelatin is a semi-synthetic biopolymer extracted from collagen, which is the most abundant protein in the body’s tissues. About 90% of the composition of gelatin is attributed to proteins, but it also contains salts and water. Its molecular structure comprises single α polypeptide chains, which may be slightly or highly cross-linked, and are generally hydrophilic. For this reason, it is a polymer that can be dissolved in water and processed to acquire different configurations, whether as hydrogels, fibers, films, scaffolds, etc. (Figure 5) [67,68,69].

An intrinsic feature of gelatin is its ability to transition between colloidal and gel states. This is justified because during the process of denaturing the original polymer, the natural triple helix configuration of collagen fibers is lost, so that they can be partially recovered when the gelatin is cooled. Between 35 and 40 °C, the colloidal solution is preserved and below this temperature range, gelation occurs as a consequence of the reconstruction of some triple helices in the molecular structure of gelatin [70].

The first step of gelatin production is to extract collagen, via heat treatments, from sources such as bones, tendons, or skin of swine and bovine animals [71]. Then, hydrolysis reactions are promoted that can be acidic, alkaline, or enzymatic. Gelatin is type A when obtained from an acid treatment, and type B when derived from a basic process [70]. In this case, it can be said that the polymer is the result of a partial degradation of collagen, considering that some chemical bonds are broken during the process, either at the atomic level (between hydrogen atoms and the triple helices), or at the intermolecular (between collagen fibrils) [72].

Regarding the swelling and dissolution of gelatin, it is assumed that these processes depend on factors such as pH, temperature and salt concentration in the solvent [67]. Compared with collagen, its derivative tends to show greater swelling and faster degradation. This is fostered by its structural configuration with less cross-helixes and the presence of hydrogen at different points in the chain. Physical or chemical crosslinkers, such as genipin, glutaraldehyde or ultraviolet radiation, can create greater stability in the biological environment [73]. 

Several studies establish the solubility [74], biocompatibility and anti-inflammatory action [68] of gelatin, through tests performed in vitro and in vivo. In addition to these attributes, the literature reports a higher antigenicity observed for gelatin compared to collagen [73,75]. Table 1 summarizes most of its benefits for tissue engineering applications. Gelatin induces an effective release of drugs and growth factors via electronic interaction when inserted into damaged tissue. Considering that the polymer is extracted from collagen, the high level of collagenase secreted at the site of injury induces the rapid degradation of gelatin molecules, producing a quick release of the factors [76]. The practice has been reported since the late 1990s when growth factors-modified hydrogels were produced for bone regeneration. For example, the release of bFGF (Basic fibroblast growth factor) incorporated in gelatin hydrogels promoted bone formation after twelve weeks in a cranial defect, while for the reference samples (without the growth factor and/or without treatment), it was only formed connective tissue at the site of the lesion [77]. Most recently, the spectrum of substances incorporated into gelatin expanded, becoming systems also applied for other tissues, such as skin, nerves, and muscles [78].

This mechanism of drug release is desirable for regenerative applications, and it is usually modulated by controlling some parameters, such as the reticulation of the gelatin structure and the isoelectric point (IEP) of the polymer [79]. Cross-linking methods are applied, in general, to prolong the gelatin degradation time, and consequently, the release rate. The IEP is defined by the processing technique of collagen and is an important factor to enable physicochemical interactions between polymeric chains, drugs, and/or molecules. In summary, if the protein or drug to be released is alkaline, acid gelatin (IEP 5.0) should be selected; for an acidic substance, the collagen derivative should be used in its basic form (IEP 9.0) [76].

In addition to its notable role in stimulating tissue regeneration, gelatin’s role in the immune system and in cancer therapy became known. Microspheres produced by Yoshimoto et al. [80] enabled the detection of the pro-inflammatory phase of macrophages, making the monitoring of the inflammatory process more detailed. For cancer use, the gelatin-based system can be applied to optimize disease monitoring, when combined with inorganic signal-emitting particles; to encapsulate tumor cells present in the blood; or to be administered directly to the tumor site, delivering growth factors that increase the treatment efficiency [78,81].

The use of gelatin nanoparticles modified by epidermal growth factors for lung cancer treatment has already been studied and can be administered directly to the lungs via inhalation [82]. Another application route is the replication of a tumor microenvironment, as was achieved by Brancato et al. [83], who combined gelatin microparticles, pancreatic cancer cells, and fibroblasts with the aim of understanding the complexity of the stromal-cancer relationship.

The expansion of 3D bioprinting technology using gelatin combined with other polymers, mainly polysaccharides, has also shown desirable results in the development of organs and other structures with predefined architectures that create an artificial cellular microenvironment [84,85]. The results of the studies by Jia et al. [86] and Lee et al. [87] evidenced the functionality of this technology, presenting the possibility of building native-like perfusable vessels and artificial cardiac microtissues with adjustable stiffness and degradation, respectively.

Due to the versatility and attributes mentioned above, there are many sectors in which gelatin can be used, including the food, pharmaceutical, cosmetic and biomedical industries [72]. In addition, the ability of the biopolymer to form composites and hybrids with other polymers and inorganic phases is added, which is widely applicable to tissue engineering [88,89,90,91]. For example, an organic-inorganic hybrid based on gelatin and silica has already been explored and shown to be versatile in tissue engineering. This is noteworthy since silica is also found in many bioactive glasses, including the pioneering Bioglass^®^ 45S5 composition. Hybrid scaffolds produced by 3D printing using gelatin and the silica precursor GPTMS (3-glycidyloxypropyl)trimethoxysilane) proved to be viable for articular cartilage regeneration, having pores of appropriate size for cartilage matrix formation (~200 μm) and close mechanical properties to the native tissue [92]. Other 3D printed gelatin-methacryloyl (GelMa)-based scaffolds with different concentrations of silanated silica (tetraethylorthosilicate (TEOS)-GPTMS) showed promise for hard tissue applications. In general, there was an increase in the properties of the GelMa-silica scaffolds compared to the control groups. The elastic modulus increased by a proportion of 12–23%, while calcium deposition was 185% higher for these samples [93]. 

Furthermore, crosslinking gelatin scaffolds with GPTMS increased the stability of the material after subcutaneous implantation in rats, prolonging the degradation time, which for non-crosslinked samples was 4 weeks [94]. A relevant observation discussed by the authors was the decrease in cell proliferation in the cross-linked scaffolds. The release of silica likely causes the cells to detach from the scaffold during the assay. Therefore, it is necessary to evaluate the content of crosslinking agent and the method used in order not to compromise the performance of the material in vivo.

Nanofibers of organic-inorganic composition (gelatin-silica) demonstrated effectiveness in carrying the drug metronidazole, in addition to exhibiting high bioactivity and antibacterial action [95]. The electrospinning technique was also used in the synthesis of fibers based on GelMa and calcium phosphate nanoparticles, which promoted angiogenesis, osteogenesis and significant mineralization in vitro, evidencing the potential of these hybrids for tissue regeneration [96]. In addition, combinations between the collagen derivative and bio-glasses and/or glass-ceramics [97,98] have been addressed in the literature, as discussed in more detail in subsequent sections.

**Table 1:** Beneficial roles and applications of gelatin in tissue engineering.

Field	Properties	References
General	Biodegradability Biocompatibility Higher antigenicity than collagen Anti-inflammatory action Hemostatic action	[70,74,75,78,79,81]
Processing	Hydrophilicity Capability to form composites and hybrids Cross-linked to increase the stability Thermo-reversibility between colloidal and gel states Higher swelling and faster degradation than collagen Different configurations (hydrogels, microspheres, fibers, scaffolds, etc.)	[67,68,69,70,71,73,82]
Drug release	Combination between substances with distinct pH Rapid release of drugs and growth factors via electronic interaction	[76,77,78,79]
Immune system	Improved monitoring of the inflammatory process Detection of the pro-inflammatory phase of macrophages	[78,80]
Cancer therapy	Delivering growth factors directly to the tumor site Encapsulation of tumor cells present in the blood Optimization of the disease monitoring	[78,80,81,82,83]
3D bioprinting	Matrix for cell culture Development of complex structures Control of the porosity	[70,78,81,82,84,85,86,87]

## 5. Bioactive Glasses (BGs)

Fundamentally, the glassy state of a substance is similar to its supercooled liquid form. It is characterized by a non-equilibrium state that tends to undergo continuous “relaxation” and has a glass transition. Glass has a predominantly non-crystalline, transparent and brittle structure that can crystallize as it undergoes specific heat treatments [99]. 

The so-called bioactive glasses, in turn, are able to bind to soft and hard tissues, are biodegradable and stimulate cellular activity responsible for promoting tissue regeneration through the release of bioactive ions [100,101,102,103]. These ions come from the oxides that form or modify the vitreous network, which are introduced into the system during synthesis in the form of precursors [104,105].

From the point of view of chemical composition, bioactive glasses can be divided into three groups: based on silicate (SiO_2_), phosphate (P_2_O_5_) and borate (B_2_O_3_). Those based on silicate became more widespread due to their excellent performance as a glass network former [106]. Other components, called network modifiers, can be implemented to add properties such as surface reactivity in a biological medium. Included in this category are the CaO, K_2_O and Na_2_O oxides [107]. Other elements, primarily transition metals, have shown therapeutic benefits such as anti-bacterial, anti-inflammatory, and other biologic responses to further aid in the medical devices’ performance [108,109,110].

Different methods used to synthesize bio-glasses are reported in the literature, including melting-quenching and sol-gel. The sol-gel method has been used for the same purpose since the 1990s when Larry Hench and colleagues observed enhanced bioactivity for such glasses. Since then, the method has become much more recognized for biomedical applications. Sol-gel synthesis shows promise in the formation of multi-component hybrids, composites and bio-glasses than those obtained through other methods [111,112,113].

The use of glasses as medical materials began in 1969 when Larry Hench obtained the first quaternary composition, which was later named Bioglass^®^ 45S5 [106,114,115]. The first approved device, registered in 1985, was for the repair of the middle ear ossicular chain using MEP (Bioglass^®^ ossicular reconstruction prosthesis) and, in 1988, the second bioactive glass-based product was produced in the form of a cone and applied to the tooth extraction site. As the device became known, the ERMI (endosseous ridge maintenance implant) was marketed in the United States [106,116]. In the early 1990s, the development of bio-glass compositions in the form of particles or granules began, which could be adapted according to the type of procedure. To facilitate clinical handling, it was common to soak the material with the patient’s blood, resulting in a pasty texture that could be injected or spread to fill the bone defect [117].

With the aim of replacing bone grafts, which generated risks of infections and mechanical failures during use, PerioGlas appeared in 1993, NovaBone in 1999 and BonAlive around the 2000s. In 2005, in the United Kingdom, Theraglass marked the first bio-glass composition modified by the incorporation of ions, which consisted of a healing gel with glass particles containing 2 mol% silver. This study concluded that low concentrations of these ions have bactericidal properties, maintaining the cell viability of the compound [118].

Most existing BG compositions currently have precursors of Si, Ca and P. Briefly, it can be said that the presence of silicon is essential for bone formation and calcification to intensify collagen formation and regulate the expression of VEGF. Calcium stimulates osteoblastic proliferation and differentiation, matrix mineralization, expression of growth factors and migration of epidermal cells for wound healing. Phosphorus, in turn, is known to stimulate the expression of essential proteins in bone metabolism [25,26,119].

In addition, the modification of bioactive glasses by using dopants has been the object of investigation in the development of these materials, in view of their relevant contribution to bone metabolism and soft tissue reconstruction [18,109,120]. In this sense, some studies report effects promoted by modifying agents, which are summarized in Table 2.

Starting from the premise that the bioactivity of bio-glasses is due to the release of ions present in the glass structure, it is important to understand the mechanisms of release, which can occur by diffusion, chemical effect, solvent, or by combined action of these factors. The first occurs when the ions pass through the pores in the matrix structure and are dissolved in the medium, while the second occurs when the phases are chemically bonded. Both mechanisms can happen naturally and simultaneously in a single system. Solvent release, on the other hand, is supposed to rely on the interference of an external agent [105].

Currently, bioactive glasses and glass-ceramics make up the emerging generation of bioactive materials, standing out for their compositional versatility. The action of different ions that stimulate cellular metabolism allows the application of these materials in the repair of bone defects, tissue regeneration, wound healing, cell support, drug delivery, etc. [1,117,157,158].

Studies published in recent years have proven that these materials can act with similar efficacy to classic orthopedic treatment in soft tissue applications. Release of SiO_4_^−4^ species aids in the remodeling of difficult-to-heal wounds and in the control of hemorrhages. These units stimulate the signaling of growth factors and proteins in addition to promoting the activation of clotting factors, processes that are essential for accelerating the proliferation of epithelial cells and reducing inflammation. In addition, biological mechanisms and recovery are facilitated due to the high surface area of nano/micro gel-derived BG particles, which considerably improves the reactivity of these particles in the medium [24,57,60].

Bioactive glass scaffolds for bone bonding applications, drug delivery, and therapeutic ion release have primarily been produced through 3D printing [159,160]. Three-dimensional printing processes such as robocasting, lithography, or selective laser sintering have been the most popular technique for manufacturing these devices [161,162]. Scaffolds produced through 3D printing have shown promise in vitro showing biocompatibility and cellular activation of VEGF proteins while also showing comparable mechanical properties to bone while tested in vivo [163,164]. Composite materials using both synthetic and natural polymers can be combined with BGs and bioceramics to produce through 3D printing techniques for even highly optimized medical devices [3,164,165,166,167,168]. 

## 6. Composites of Gelatin/BG for Tissue Engineering

Modification of gelatin matrices with bioactive glasses improves mechanical stability, while increasing the rate of material degradation in a physiological environment. The first characteristic is caused by the presence of bio-glass particles and/or fibers in the polymer chain, which act as a reinforcement, making it more rigid [169,170]. The second property is due to the ion exchanges produced by BG in aqueous media, which increases the hydrophilicity of the composite as a whole and accelerates the degradation kinetics [3,171].

Composites can be synthesized through different pathways, resulting in fibers, films, microspheres, hydrogels, and scaffolds. Scaffolds contribute significantly to cell interaction as there is high surface area for cell proliferation and attachment. This causes increased ECM formation, allowing regenerated tissue to replace the decomposed scaffold [172]. In this context, structures can be obtained by several techniques, including the freeze-drying method [173], electrospinning [174], foaming [175], sol-gel [176], 3D printing [177], or by the use of pyrogenic agents such as PMMA [178]. More recently, 3D/4D printed scaffolds have become more widespread in the scientific environment due to the greater precision provided by the technique for controlling material properties, such as porosity and pore size, degradation profile, drug release and others, thus being able to create structures similar to living tissue [165,179,180,181].

### 6.1. Bone Engineering/Repair

The incorporation of BGs in gelatin matrices promotes more effective mineralization when compared to the gelatin used alone [19,21,182,183]. Another important aspect is the chemical similarity of these composites with the natural mineral matrix. Therefore, the bio-glass tends to reinforce the mechanical stability of the polymer, while it provides the desired tenacity and elasticity for the bone tissue [22,41,104].

Since the early 2000s, scaffolds with high porosity have been prepared using chitosan, gelatin and 55SiO_2_–40CaO–5P_2_O_5_ (mol%) nanoparticles, with potential application in both bone and cartilage tissue repair [15,16]. Using simply the lyophilization approach, pores ranging from 150 to 300 μm were easily produced, allowing cell migration and fixation. After 14 days of in vitro bioactivity test, MG-63 cells established cellular bridges between the pores of the structure, and a uniform deposit of apatite formed on the surface of the scaffolds [16]. 

Later, Sarker and his collaborators [184] produced hydrogels based on alginate, gelatin and bioactive glass Bioglass^®^ 45S5 (45SiO_2_–24.5CaO–24.5Na_2_O–6P_2_O_5_ mol%) in order to generate scaffolds capable of stimulating bone mineralization. In this sense, it was observed that even in compositions with only 1% by weight of melt-derived Bioglass (~2 µm particles), there was apatite formation and significant cell proliferation along the morphology of the scaffolds produced by lyophilization. The authors emphasized that the gelatin and bio-glass combination provided the optimum support for cell growth as well as a greater spread of cells between the pores.

Since then, different studies have shown positive results for hydrogels, scaffolds and coatings based on gelatin and bioactive glass targeted for applications in bone tissue repair, exhibiting improved mechanical properties and bioactivity [185]. Nanofibrous scaffolds based on bioactive glass 62.7SiO_2_–33.2CaO–4.1P_2_O_5_ (mol%) and gelatin were synthesized via a template-assisted sol-gel process and generated a greater proliferation of osteoblasts and secretion of the enzyme alkaline phosphatase (ALP), compared to the polymer in its pure form. As for the compressive strength results, the nanofibers had an increase, on average, of 128.5% in relation to the value obtained for isolated gelatin [176].

Abd El-Aziz et al. [20] added 5% by weight of 55SiO_2_–24CaO–6P_2_O_5_–15B_2_O_3_ nanoparticles (mol%) in gelatin matrix, resulting in scaffolds with notably increased bioactivity and durability. Hybrid fibers of bioactive glass 70SiO_2_–25CaO–5P_2_O_5_ (mol%) and GPTMS functionalized gelatin promoted the formation of apatite crystals with only 12 h of immersion in SBF. In addition, the fibrous architecture scaffolds stimulated the fixation and proliferation of osteoblastic cells of the MC3T3-E1 type and had a higher ductility (from 63 ± 2 to 168 ± 14%) and tensile strength (from 0.5 ± 0.2 at 4.3 ± 1.2 MPa), compared to unmodified polymer [186].

Zheng and his collaborators [187] produced hydrogels using GelMa and bio-glass 40SiO_2_–45CaO–15P_2_O_5_ (mol%), for which cell adhesion, proliferation and osteogenic differentiation were visualized and confirmed by the expression of ALP in the samples. Another research involving gelatin scaffolds with ~100 µm pores and neutral pH BG (54.2SiO_2_–35CaO–10.8P_2_O_5_ mol%) with particles ≤ 10 µm resulted in good in vitro mineralization and biocompatibility. The samples also exhibited mechanical properties suitable for application in dental pulp regeneration, with a stiffness of 50–60 kPa [188].

Some studies developed from the addition of bio-glass nanoparticles 64SiO_2_–31CaO–5P_2_O_5_ (mol%) synthesized via sol-gel in gelatin matrix registered a Young’s modulus of 50–80 MPa [189,190] and a compressive strength of 2.8–5.6 MPa [182,191]. The composite displayed an average pore size of 200–500 µm, obtained by lyophilization. But in general, despite achieving stability approaching that of cancellous bone, the reported compressive strength for gelatin/bio-glass scaffolds is still far from that estimated for cortical bone.

In addition to the effects of the interaction between the two species, modification of bioactive glasses by dopants interferes with the mechanisms that occur at the material/polymer interface, including the degradation rate, biological properties, mechanical and chemical [105]. For example, the incorporation of Sr-modified BG nanoparticles (SiO_2_–CaO–P_2_O_5_) into the gelatin matrix accelerated the degradation rate of the scaffolds, which may be associated with the replacement of Ca^2+^ ions by Sr^2+^ ions. Because they are larger, these ions favor the disorder of the glass lattice and, consequently, facilitate its rupture [150]. Furthermore, increasing the percentage of BG-Sr improved the compressive strength and elastic modulus of the scaffolds produced by freeze-drying.

Scaffolds prepared with a bioactive glass (150 nm particles on average) doped with manganese (50SiO_2_–35CaO–10P_2_O_5_–5MnO in mol%) and gelatin, with porosity > 80% exhibited higher cell viability in vitro and a compressive strength of five times higher than the result envisaged for glass only [147]. 

The combined use of Mg and Zn (SiO_2_–CaO–P_2_O_5_–MgO–ZnO) promoted antibacterial activity, associated with zinc, as well as stimulated type I collagen expression and alkaline phosphatase (ALP) activity, as a result of the presence of magnesium [144]. On the other hand, the use of ZnO whiskers was reported by Guo et al. [154], who mixed it with the bioactive glass 60SiO_2_–36CaO–4P_2_O_5_ (mol%) for later addition in a gelatin solution. In this case, the scaffolds with only 2% by weight of ZnO increased the proliferation of rat mesenchymal stem cells (rMSCs) and optimized the mechanical performance.

**Figure 6:**
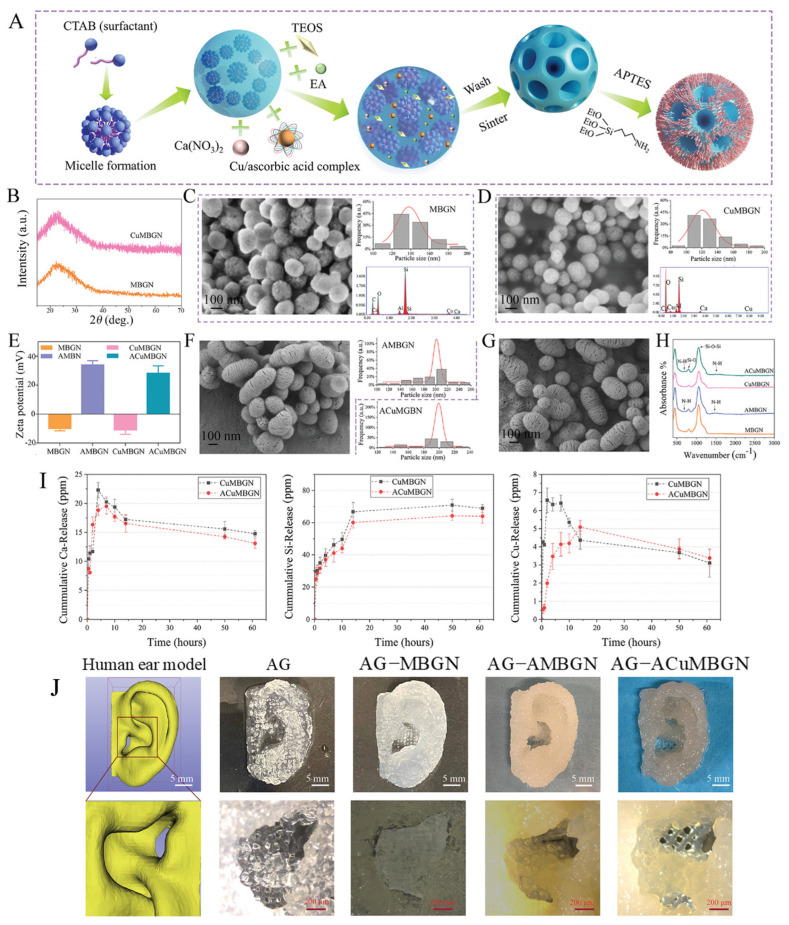
(**A**) Schematic diagram of the formation and surface functionalization mechanisms of MBGNs. (**B**) Amorphous structure of the synthesized Cu-doped and Cu-free MBGNs confirmed by XRD. SEM images, particle size, and element analysis of (**C**) MBGNs and (**D**) CuMBGNs. (**E**) Zeta potential of MBGNs and CuMBGNs before and after surface amination. SEM images and particle size analysis results of (**F**) AMBGNs and (**G**) ACuMBGNs: A signifies amination. (**H**) FTIR spectra of MBGNs and AMBGNs before and after surface amination. (**I**) Calcium, silicon, and copper ions release from CuMBGNs and ACuMBGNs during 3 days’ immersion in Tris-buffer solutions at 37 °C. (**J**) Photographs of printed, gelatin, alginate, and mesoporous BG model ear structures and their enlarged images. Adapted from Zhu et al. [192].

Zhu, et al. [192] formulated 3D-printed scaffolds using gelatin, dialdehyde alginate and Cu-doped mesoporous bioactive glass nanoparticles (MBGNs), the latter being synthesized via sol-gel starting from the base binary composition of 85SiO_2_–15CaO (mol%). The mechanisms by which MBGNs were synthesized and underwent surface functionalization are shown in Figure 6A. Using cetrimonium bromide (CTAB) micelle as a template can self-assemble bioactive glass (BG) precursors on the surface of the template due to hydrogen bonding during the sol-gel process to generate mesoporous spherical particles. Once the mesoporous MBGNs have been calcined and washed, the CTAB can be removed. Then, 3-aminopropyl triethoxy silane (APTES) was allowed to react with the glass surface in toluene at a refluxing temperature while being stirred constantly to functionalize MBGNs surface by amino groups. The condensation of MBGNs’ surface hydroxyl groups (silanol groups) and APTES’ ethoxy groups can be accelerated by heating the mixture to high temperatures. Figure 6B displays the X-ray diffraction analysis (XRD) patterns of MBGNs and Cu-doped MBGNs following calcination at 700 °C, providing further evidence of the amorphous nature of these glass nanoparticles. Particle structure is unaffected by the incorporation of Cu/ascorbic acid complex. Field emission scanning electron microscopy (FE-SEM) images of both produced MBGNs and CuMBGNs (Figure 6C,D, respectively) revealed consistent sphere-like morphology with mesopores on the surface. Statistical examination of the images revealed that the MBGNs and CuMBGNs had sizes of 142 ± 17 and 128 ± 20 nm, respectively. Energy dispersive X-ray spectroscopy (EDS) confirmed a Cu concentration of 2.5 at% in CuMBGN. Figure 6E shows that after surface amination, particles’ zeta potentials increased dramatically (Figure 6E). Figure 6H shows the Fourier transform infrared spectroscopy (FTIR) analysis, which revealed that APTES-treated particles exhibited more NH bonds than non-aminated MBGNs (695 cm^−1^ vibrations and 1570 cm^−1^ bending mode). This suggests that the particle surfaces have been produced with aminopropyl moieties, which may present opportunities for imine couplings with aldehydes. SEM analysis of the morphology of the aminated particles revealed a larger size distribution and a larger mean diameter compared to the nonaminated particles (Figure 6F,G). The authors found that both CuMBGNs and ACuMBGNs release inorganic ions of Si, Ca and Cu in a therapeutic range (Figure 6I). Amine-functionalization of CuMBGNs decreases calcium and silicon release throughout the test. Compared to non-functionalized CuMBGNs, amine functionalization considerably slowed copper release in the first 24 h of immersion in Tris buffer. The results evidenced the improvement of the material properties after the addition of both MBGNs to composites, such as the increase in ALP activity and apatite deposition in vitro in just 3 days for composites. The 3D-printed gelatin, alginate containing MBGNs model ear structures and their enlarged images are shown in Figure 6J.

As for antibacterial properties, Zheng et al. [133] were successful with a Cu-modified bioactive glass (95SiO_2_–2.5CaO–2.5CuO mol%). The dopant increased the osteogenic activity of the scaffolds and increased the antibacterial effect. The combination of Ag and Sr was reported by Aqib et al. [121], who used mesoporous 50SiO_2_–10P_2_O_5_–34CaO–5SrO–1Ag_2_O (mol%) glass, verifying an increase in the bioactivity of chitosan/gelatin scaffolds and an antibacterial action against gram-negative species induced mainly by silver. On the other hand, the presence of these ions reduced the cell viability, but the combination with strontium mitigated the cytotoxic effect caused by Ag_2_O.

Antibacterial properties were also seen for pure BG compositions, that is, without the presence of dopants. For example, Yazdimamaghani, et al. [193] synthesized 63S bio-glass nanoparticles (63SiO_2_–4P_2_O_5_–31CaO mol%) to compose the gelatin scaffold. The samples promoted better viability of human mesenchymal cells (hMSC) and showed antibacterial effects against *Escherichia coli* and *Staphylococcus aureus*. However, the growth inhibition was more pronounced when the authors modified the scaffolds with silver nanoparticles.

Nanofibers of gelatin, polycaprolactone (PCL) and bio-glass 45SiO_2_–24.5CaO–6P_2_O_5_ (mol%) were produced by electrospinning in the study of Elkhouly, et al. [194]. The authors reported that the presence of sol-gel produced BG made the scaffolds more hydrophilic and increased swelling. Furthermore, bioactive glass nanoparticles improved tensile strength, elastic modulus and ductility compared to samples without BG. This is desirable to increase the proliferation of osteoblasts since the stiffness of the material influences this mechanism. A layer of hydroxyapatite covered the surface of the composites after 14 days. For the hybrid scaffolds produced by Houaoui, et al. [195], containing gelatin-GPTMS and silicate (13-93) and borate (13-93B20) bio-glasses obtained by melting (particles < 38 µm), the precipitation of hydroxyapatite after two weeks of immersion in PBS was observed.

A recent work focused on hydrogels based on GelMa and 45SiO_2_–24.5CaO–6P_2_O_5_ (mol%) BG, shows a significant increase in the proliferation and proliferation of osteoblastic cells. In vitro cell viability was 75.6 ± 3% for the scaffold without BG and grew to 98.6 ± 0.9% when adding 5 wt.% bio-glasses—also increasing ALP activity 2–3 times higher for the highest concentration samples. The mechanical properties (modulus of elasticity and compressive strength) were also 1.8–2 times higher with only 5% of gel-glass nanoparticles. Unlike other studies discussed in this section, there was a decrease in the degree of swelling and the rate of degradation of the scaffolds [196].

The success of gelatin/BG composites in animal tests has also been addressed in the literature. Bioactive gelatin/glass 64SiO_2_–31CaO–5P_2_O_5_ (mol%) scaffolds demonstrated considerable cell viability and in vitro bioactivity and were implanted into a 6 mm rat calvaria defect, for which they demonstrated the ability to promote new bone tissue formation. The sol-gel method was selected for the synthesis of BG nanoparticles and the lyophilization and lamination techniques to produce porous composites. It is estimated that tissue growth increased between 4 and 12 weeks, a period in which scaffold resorption and infiltration of osteoprogenitor cells and blood vessels occurred simultaneously [197].

**Figure 7:**
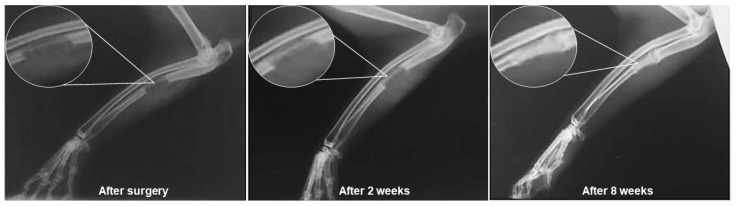
Radiographs of the ulnar segment defect immediately after surgery, after 2 weeks and 8 weeks. Adapted from Hafezi et al. [198].

Among the other in vivo studies reported, Hafezi et al. [198] described the result of the implantation of a gelatin/nanopowder scaffold of 64SiO_2_–31CaO–5P_2_O_5_ (mol%) glass in the ulna of rabbits. A mineralization was observed that started at the bilateral ends and significantly diffused towards the center of the implant after 8 weeks. Through radiographic evaluation of the defect, the authors verified that at the end of the second week of implantation, there was already a well-defined extension of the defect along the ulna, which was gradually consolidated by the bone healing process (Figure 7).

Kargozar et al. [191] carried out experiments with gelatin-based scaffolds and glass nanoparticles of the same composition but seeded with rat bone marrow cells. The system was inserted into a critical-sized defect in the animals’ calvaria and promoted effective osteogenesis and healing between 4 and 12 weeks after the procedure. However, at the end of 12 weeks, no sign of newly formed bone tissue was seen for the gelatin/bio-glass control samples without cell seeding. In this, the authors could only verify the presence of a fibrous tissue after 4 weeks, and a bond formed between the host bone and the implant, which was expected due to the inherent ability of bioactive glasses to bond to the calcified tissue.

A system composed of gelatin and ternary SiO_2_–CaO–P_2_O_5_ glass produced by the sol-gel method was also subjected to a seeding with osteoblast lineage and inserted into critical-sized defects of rat calvaria [199]. After one week of implantation, the presence of inflammatory cells and bone tissue on the edges was detected in the defects filled with scaffolds. At the end of 30 days, for samples seeded with osteoblasts, bone formation had already started to develop towards the central zone of the defect, as well as the presence of collagen. In these samples, the neoformed tissue developed over 90 days, while there were still remnants of the scaffold in the degradation process.

Covarrubias, et al. [200] applied lyophilization to produce chitosan-gelatin/BG 58SiO_2_–40CaO–5P_2_O_5_ (mol%) scaffolds and evaluated their performance through bioactivity and cell viability assays using dental pulp stem cells. After confirming the effectiveness of the composite in vitro, they were evaluated for the ability to promote bone regeneration in critical-sized femoral defects in rats. The authors found an increase of ~80% in the amount of new bone tissue formed after 8 weeks of implantation of scaffolds containing 5% by weight of bioactive glass nanoparticles synthesized via sol-gel.

Other researchers have developed tantalum (Ta) scaffolds coated with the gelatin/bio-glass system 60SiO_2_–36CaO–4P_2_O_5_ (mol%), achieving interesting results from this association. The in vivo assay was performed on the femur of rats and evaluated for 8 weeks after insertion of the material. For the polymer implant without modification, only the presence of fibrous connective tissue was verified, as well as for the metal control sample. On the other hand, sol-gel-derived bio-glass scaffolds (400–500 nm particles) induced bone formation in the cavity, demonstrating significant osseointegration in the initial phase of implantation [201]. After 6 weeks, the presence of newly formed bone tissue was observed in composites made with 5 wt.% of bioactive glass 45S5 and gelatin matrix that implanted in ectopic bone defects in rats [202]. 

Furthermore, the authors found a possible relationship between the increased rigidity of the lyophilized scaffolds and the increased cell proliferation indicated in in vitro tests using human mesenchymal stem cells (hMSCs). They considered the hypothesis that the improvement of mechanical properties had induced the cells to be osteogenic, and not chondrogenic, differentiation, corroborating what was discussed by Elkhouly et al. [194].

In a study by Dai et al. [132], the sol-gel method was used to develop hydrogels modified by a bioactive copper-doped glass (BG-Cu), with the goal of examining its function as a stimulant of human umbilical vein endothelial cells (HUVECs) and bone mesenchymal stem cells of mice (BMSCs) in vitro. The hydrogels were transformed into scaffolds by 3D printing and inserted into bone defects in rats. This demonstrated that the addition of only 1% by weight of micro/nano BG-Cu particles was sufficient to promote significant vascularization and osteogenesis.

Another example of the application of the 3D printing technique in the development of scaffolds applied to bone regeneration was addressed by Wu et al. [180]. This study combined gelatin, sodium alginate and 80S mesoporous bio-glass (80SiO_2_–16CaO–4P_2_O_5_ mol%). The bioactive glass particles, resulting from an evaporatively induced self-assembly (EISA) were ground to 74 µm. In general, the scaffolds had a porosity of about 80%, similar to human cancellous bone, and exhibited high biocompatibility, biodegradability and osteogenesis.

Strontium-doped gelatin/bioactive glass scaffolds (SiO_2_–CaO–SrO–P_2_O_5_ mol%) were tested on rabbit calvaria bone defects. After 4 weeks, the defects filled with the gelatin/BG-Sr group showed bone tissue formation and the presence of mature collagen, so that over 8 and 12 weeks there was a healing of the regenerated bone, while in the control group the defect was filled almost entirely by fibrous tissue. For the gelatin/BG SiO_2_–CaO–P_2_O_5_ (mol%) group, bone formation started only after 8 weeks [152].

**Figure 8:**
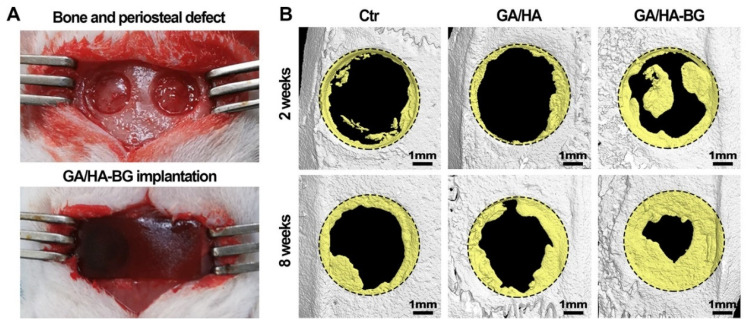
Images of the critical-sized bone defect and implantation of the biomimetic periosteum in vivo (**A**); 3D reconstruction of the defect areas at 2 and 8 weeks (**B**). Adapted with permission from Yang et al. [203]. Copyright 2022 American Chemical Society. Ctr, GA, HA and BG indicate control, gelatin, hyaluronic acid and bioactive glass, respectively.

Recently, membranes derived from gelatin-hyaluronic acid (HA) hydrogels and mesoporous 60SiO_2_–36CaO–4P_2_O_5_ (mol%) bio-glass were tested in critical-sized bone defects without periosteum in mice (Figure 8). The pure polymer membranes degraded too quickly allowing the lesion zone to fill with fibrous tissues and in turn making bone regeneration difficult. On the other hand, BG-modified membranes attracted surrounding stem cells to the defect, which differentiated into osteoblasts and spread from the edges to the multicentric region. In addition, these samples induced revascularization and degraded more slowly, enabling effective bone regeneration within 8 weeks [203].

Gelatin/BG composites also demonstrate a promising alternative for drug release [204,205,206,207]. Pajares-Chamorro et al. [122] applied gelatin scaffolds modified by Ag-doped 58S bioactive glass (58SiO_2_–33CaO–9P_2_O_5_ mol%) (particles smaller than 20 µm) in defects similar to the one mentioned above. The authors highlighted a greater filling of the defect with newly formed bone tissue for the gelatin/BG-Ag system. Furthermore, the bio-glass was able to carry the drug vancomycin at concentrations of 0.3–1 mg/mL, showing that the combination of the drug used, and the bactericidal agent (Ag) represent a promising alternative to regenerate and protect the bones against infections.

Another system synthesized by Govindan et al. [151] demonstrated the ability to release the drug ciprofloxacin in a sustained and prolonged manner. It consisted of 45P_2_O_5_–24CaO–21Na_2_O–5SrO–5Fe_2_O_3_ (mol%) BG obtained by melting, with particles of ~40 µm, added in a gelatin matrix, with a porosity greater than 70% and pores diameter of 100 and 500 µm.

### 6.2. Soft Tissue Engineering/Repair

In addition to the potential for orthopedic applications, the combination of gelatin and bioactive glass is promising for soft tissue repair [2,27,28]. When obtained in the form of high porosity scaffolds, these composites function as a barrier that protects the lesions from the action of microorganisms while also reducing exudates and the inflammatory response. These structures can also promote hemostatic and angiogenic effects that favor tissue granulation and healing [24,26]. Regarding the processing of hydrogels, gelatin is one of the best candidates as a matrix for bioactive glasses, as it is possible to combine the bioactivity of these particles with the thixotropy of the polymer, which in its gel state can acquire greater fluidity at temperatures close to that of the body (37 °C) and achieve a better fit at the wound site [69]. 

The synthesis of hybrid materials containing gelatin, 58S bioactive glass and graphene oxide (GO) for application in tissue engineering was addressed by Zeimaran, et al. [208], who used sol-gel and gas foam techniques to produce ~170 µm pores, suitable for revascularization. Hybrid samples were produced from the functionalization of gelatin with GPTMS, followed by the addition of the 58S-GO mixture. In the in vitro cytotoxicity test with human mesenchymal stromal cells derived from adipose tissue, the scaffolds were biocompatible and stimulated cell adhesion and proliferation.

Gelatin, hyaluronic acid and bioactive glass nanocomposites were developed and subjected to biological tests, including skin irritation and acute toxicity studies. The bio-glass of composition 60SiO_2_–35CaO–5P_2_O_5_ (mol%) synthesized by the sol-gel method was added (5% by weight) in a gelatin-HA solution, and scaffolds with pores of 50–500 µm were obtained by lyophilization. In vivo tests were performed on rabbits for skin irritation analysis and mice for cytotoxic analysis. In summary, 3 days after the dermal application of the product, no evidence of toxic effect was detected in the implant region in animals. Corroborating these findings, the organs of mice examined at necropsy showed no macroscopic lesions caused by the treatment, and the hemolysis rate was 1.11%, indicating that the material is biocompatible [209].

In another survey by Zhou, et al. [210], the potential of scaffolds of compositions similar to the one mentioned above was evaluated, this time from an application perspective for soft and hard tissues. The samples were obtained by lyophilization and a reduction in pore size was observed when increasing the concentration of BG from 5 to 30% by weight. At the same time, the incorporation of bioactive glass nanoparticles resulted in less swelling, which contributes to better mechanical stability, but may be unfavorable to cell adhesion. Nevertheless, in the in vitro cytotoxicity assay, the gelatin-hyaluronic acid/BG composites slightly increased cell viability compared to the polymeric scaffold and the negative control used in the test. A likely reason for this is the ionic release mechanism of the bio-glass, which promotes alkalization of the medium, mainly attributed to Ca^2+^ ions, thus stimulating the apoptosis of fibroblast cells.

Other work was published for application simultaneously in soft and hard tissues. In this case, the addition of bioactive glass to the polymeric matrix accelerated in vitro biodegradation and the bioactivity was observed only after 14 days, which the authors justify due to the low concentration of BG. However, considering that the implant would be in contact with cartilaginous tissue and not with bone, it is possible that the material promotes the expected regenerative response [30]. More specifically, it consisted of a coating composed of gelatin, alginate, and 45S5 bio-glass developed for titanium (Ti) substrates applied to osteochondral defects. The coatings were obtained by adding glass powder (d50 = 4.5 µm) to the polymeric solution, using a concentration of 5 wt.%, and after gelation, a lyophilizer was used to produce the porous structures [30]. The gelatin-alginate/BG system obtained a greater elastic recovery compared to the others, which is important for cartilage applications.

Using the electrospinning technique, gelatin-chitosan/BG nanofibers 30SiO_2_–27CaO–20B_2_O_3_–4P_2_O_5_–1.5CuO–1ZnO–3K_2_O–9Na_2_O (mol%) were produced. The bio-glass was prepared by sol-gel, with a distribution particle size of 840–1660 nm. The tensile strength of the fibers increased by almost 150% after the addition of 15% by weight of BG. Furthermore, these samples degraded within 4 weeks after implantation in subcutaneous regions of mice, demonstrating the potential for application in chronic wound healing. Interestingly, the nanofibers containing the highest percentage of bioactive glass (12 and 15%), which had a more accelerated biodegradation profile, were those that had the highest contact angles (84.9 and 94.6°, respectively) [26].

Afghan et al. [25] synthesized hydrogels based on gelatin, polycaprolactone (PCL) and BG 60SiO_2_–30CaO–8P_2_O_5_–2Ag_2_O (mol%) derived from sol-gel (particle size < 50 µm), which were converted into scaffolds for in vivo analysis. These were inserted into subcutaneous lesions generated in mice and monitored for 21 days. The authors observed that wounds treated with the polymer matrix alone had a slower rate of closure, which increased with the incorporation of silver-doped bio-glass. In addition, PCL-gelatin scaffolds exhibited an intense inflammatory response, little or no vascularization and granulation. In contrast, samples containing BG could induce tissue granulation and proliferation of fibroblast cells after the first week of implantation. It is suggested that such effects occurred due to the upregulation promoted by Si ions in the expression of VEGF, as well as the antimicrobial properties conferred by the Ag dopant.

**Figure 9:**
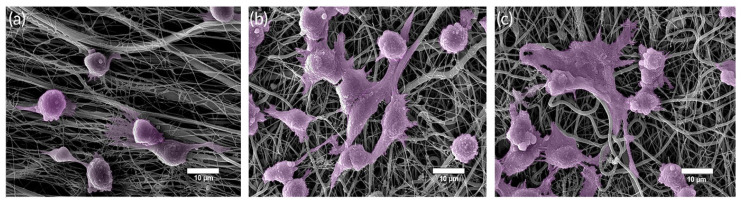
Images obtained by scanning electron microscopy (SEM) of the scaffolds used for 24 h culture of fibroblasts: chitosan, gelatin and PEO matrix (**a**), polymeric matrix modified by BG (**b**) and polymeric matrix combined with BG-Ag (**c**). Adapted from Sharifi et al. [23].

Sharifi et al. [23] reported the use of silver, obtained the bioactive glass 45S5-Ag and incorporated it into scaffolds based on gelatin, chitosan and polyethylene oxide. Biological tests showed antibacterial activity against gram-positive and gram-negative species for samples containing BG-Ag (~36 nm particles), in addition to an improved regenerative response. To evaluate the effect of the material on wound healing, implantations were performed in the excisional wounds of mice. Between days 3 and 7, the glass-modified scaffold had already induced a considerable reduction in wound area (6.04 ± 0.64 mm^2^) compared to the control group (9.12 ± 0.85 mm^2^). After 21 days, it was possible to infer that the biodegradation of the implanted scaffolds occurred at a rate proportional to the speed of skin regeneration. Among the other results presented, it is worth noting that adding bioactive glass to the polymer matrix recruited fibroblast cells that were fixed and spread within the scaffold (Figure 9), which contributed extensively to skin healing.

**Figure 10:**
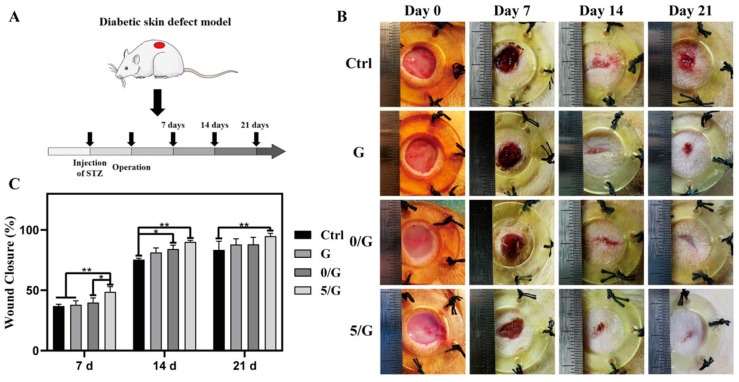
Wound healing in an in vivo study: (**A**) Skin wound model and flowchart; (**B**) Representative images of the wound area with different treatments and time periods (0, 7, 14 and 21 days); (**C**) Quantification of wound closure rate of different treatment groups (*n* = 5). * *p* < 0.05, ** *p* < 0.01 [27].

**Figure 11:**
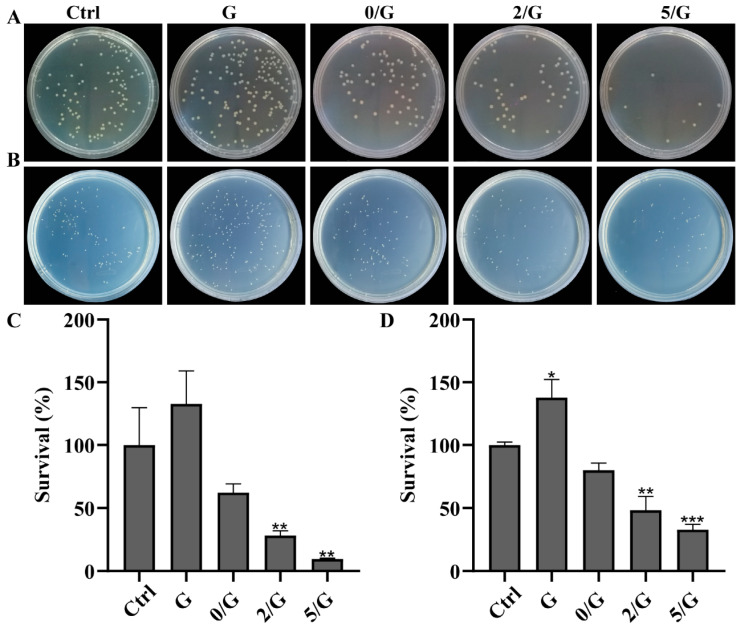
Antibacterial activity evaluation of gelatin/Ce-BG based hydrogels: GelMa (G), 0% Ce-BG/GelMa (0/G), 2% Ce-BG/GelMa (2/G), and 5% Ce-BG/GelMa (5/G). (**A**,**B**) Photographs of *E. coli* and *S. aureus* grown on agar plates. (**C**) Survival percentage of *E. coli* with different treatments (*n* = 3). (**D**) Survival percentage of *S. aureus* with different treatments (*n* = 3). * *p* < 0.05, ** *p* < 0.01, *** *p* < 0.001 [27].

The modification of bioactive glasses with cerium was also investigated. In the study involving the synthesis of gelatin hydrogels/BG-Ce nanoparticles, diabetic rats were used to monitor wound closure for 21 days. At the end of the experiment, it was observed that the addition of 5 mol% of Ce in the gelatin-bio-glass SiO_2_–CaO–P_2_O_5_–CeO_2_ system promoted a closing rate of 94.85 ± 2.33%, while the compositions of polymer alone and gelatin/BG exhibited an average rate of 88.50 ± 5.89% (Figure 10). The control group, in turn, reduced 85.43 ± 6.41% of the wound area. The presence of the dopant also conferred an antibacterial effect against *E. coli* and *S. aureuse*, which is required for hydrogels applied to chronic wounds, a category in which diabetic patients fall [27]. Figure 11 shows the antibacterial performance of such composites.

Composites’ regenerative potential in muscular and neurological systems was investigated in addition to their application in treating skin wounds. Barabadi et al. [29] produced scaffolds of gelatin, collagen and BG 45S5 nanoparticles for an in vitro study using endometrial stem cells of the cardiomyocyte lineage. Cell growth and fixation were observed in the scaffolds, which had pores with an average diameter of 373 µm. A significant increase in VEGF secretion in the bio-glass sample led to more expressive angiogenesis. Koudehi et al. [17], on the other hand, tested composites based on gelatin and bioactive glass 64SiO_2_–5P_2_O_5_–26CaO–5MgO (mol%) (particles of 20–50 nm) in sciatic nerves of mice. Three months after surgery, the implants presented a result comparable to that of the control groups, promoting the structural and functional recovery of the tissue.

A similar work was published in which gelatin and bio-glass scaffolds of the same composition modified with silver nanoparticles were developed. The authors confirmed the antibacterial activity of the samples. They performed biocompatibility assays using 0.0007 µL of Ag, minimizing the risks of the cytotoxic effect associated with the concentration of these ions. In addition, it was possible to verify the proliferation of fibroblasts around the scaffold after 3 days [28].

Sun et al. [211] also studied the repair of sciatic nerve defects by using bioactive gel-glass added to a system composed of GelMa and type I collagen. The porous structures were generated by lyophilization in the form of a stent suitable to be implanted in 10 mm nerve defects of mice. Previously conducted in vitro tests confirmed the material-induced biocompatibility, adhesion, proliferation, and cell differentiation. Then, by means of an in vivo assay, it was possible to identify a significant presence of nerve fibers resulting from regeneration guided by the composite stent, whose result was similar to that obtained by autologous nerve transplantation.

Similar to what was previously discussed about bone tissue repair, gelatin/bio-glass composites can be used for controlled drug delivery. This was discussed by Rivadeneira et al. [212], which inserted micrometric particles (5–10 µm) of 45S5 glass into a gelatin-starch matrix, which was later modified with vancomycin hydrochloride and dried to obtain a film. The authors recorded a higher rate of drug release for the BG-containing samples, as well as a more accelerated in vitro response, which occurred in a much shorter time than required for general skin wound healing (average 4 weeks). The authors also discuss the possibility of controlling the degradation rate of the composite through use of crosslinking agents. This is highly relevant and should be investigated, considering that the cell viability of the samples prepared in this study was significantly lower than the control. Controlling the rate of degradation reduces the concentration of medicines, which may improve cell viability. Another interesting point was the antibacterial activity, observed only when the antibiotic was present.

**Figure 12:**
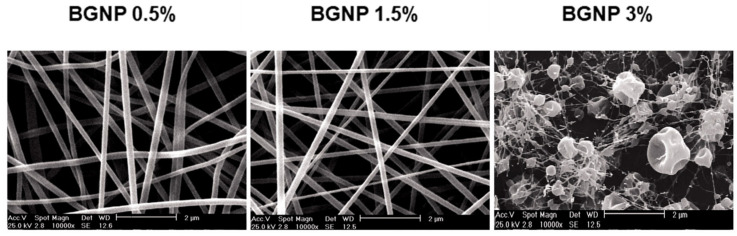
Images obtained by SEM of chitosan-gelatin/BG nanofibers with different concentrations of glass nanoparticles. Adapted from Shamosi et al. [146].

Gelatin-chitosan/bioactive glass nanofibers (Figure 12) were produced by the electrospinning method and inserted into a culture of hEnSCs (human endometrial stem cells) in order to observe the inductive effect produced by the composites in the differentiation of these cells into endothelial lineages. For this, a bio-glass nanopowder (BGNP) SiO_2_–CaO–MgO–P_2_O_5_ was synthesized, which was inserted (0.5–3 wt%) in a gelatin-chitosan solution. Cytotoxicity and cell adhesion tests revealed greater viability, proliferation, fixation and differentiation of cells grown in vitro on gelatin-chitosan/BGNP substrate, compared to controls and pure polymer nanofibers. Such results mimic the desirable effect that these nanofibrous scaffolds can generate on wound healing, angiogenesis and tissue regeneration [146].

The 3D bioprinting technique was also used in the production of scaffolds based on alginate-gelatin hydrogel modified with bioactive glass 13-93B3 (53B_2_O_3_–20CaO–12K_2_O–6Na_2_O–5MgO–4P_2_O_5_ mol%) evaluated for viability in the culture of ASC cells (mensechymal stem cells) [213]. The addition of glass took place in two ways: indirect, after dispersion in PCL; and direct, when only particles (~20 µm) were added into the polymeric hydrogel. The scaffolds had an average pore size of 309.5 ± 20 µm and their mechanical properties were improved when the bio-glass was added directly to the alginate-gelatin matrix, compared to the alginate-gelatin/PCL/13-93B3, alginate samples, and gelatin, respectively.

The cell viability of the composites was higher for the direct method of adding bioactive glass, which corroborates the discussions about the angiogenic capacity of the glass used in the research. However, a reduction in viability was reported for the scaffolds in general after 7 days, which may be associated with two reasons: the stability of the hydrogel decreases over time, due to the greater fluidity of gelatin when acclimated to 37 °C; the pH of the culture medium increases to 8.8 within this period due to the rapid dissolution of 13-93B3 particles, negatively affecting the metabolism of ASC cells [213]. 

The toxicity of the polymer matrix solvent might be a reason for decreasing cell viability. It could be evidenced in the work developed by Kolan et al. [214], who produced gelatin-alginate/PLA/13-93B3 scaffolds and performed tests on ASC cell culture from adipose tissue. Cell viability was slightly lower (~60%) in the innermost layers of the scaffold, specifically in the regions close to the PLA filaments. It was also observed that the bio-glass release rate was twice as high for samples whose printing was solvent-based (14 days), compared to the melt-based (28 days), with the former being the most recommended for tissue regeneration and drug delivery.

Another recent research developed from the 3D printing of gelatin-hyaluronic acid/BG 45S5 scaffolds was published by Bertuola et al. [215]. The bioactive glass was produced by conventional melting, reaching a particle size of 300 nm–15 µm, and added to the polymeric matrix with concentrations ranging between 2 and 8 wt.%. An increase in the modulus of elasticity associated with the bio-glass content was observed, which approached that of the skin, while the rheological properties fluctuated.

Viscosity increased for samples containing up to 4% BG and decreased when more than 6% by weight was added, which may indicate that this is the maximum content of bioactive glass supported in the polymeric network, above which flow occurs. This is an important property to be considered in studying these composites, as it determines the stability of the union between the phases. From a biological point of view, all samples proved to be biocompatible, but what drew attention was the greater roughness found on the surface of gelatin-hyaluronic acid/BG scaffolds, which is evidence that the greater reactivity induced the deposition of organic materials and inorganic substances, which is desirable for soft and hard tissue implants [215].

## 7. Challenges and Opportunities

Healing large bone and soft tissue injuries remains one of the most challenging issues. Although single materials such as bioactive glasses and gelatin have advantages in tissue engineering, restrictions of these single materials pose significant barriers, as discussed in this article. The main drawbacks of a single scaffold are brittleness, delayed degradation of BGs, weak mechanical strength, and rapid degradation of gelatin. To address these issues, composite scaffolds made of these two materials are being developed for tissue engineering applications. Despite further studies into producing such composites, their interaction with stem cells or biofactors and in vivo performance is critical and warrants additional investigation [48,50].

Gelatin is biocompatible, biodegradable, and inexpensive, with minimal antigenicity; however, its high biodegradability and poor mechanical qualities limit its applications. To achieve better outcomes in applications, chemical modifications or the fabrication of composites should often be conducted. Recent advancements in cell biology and materials science have made tissue engineering a probable treatment strategy. As such, the increasing demand is for porous biodegradable bone scaffolds, primarily for applications requiring great mechanical strength during bone repair. This is a significant design difficulty since increased porosity reduces scaffold strength [49].

Osteoconductive/inductive BGs cause relatively effective bone regrowth. These materials are used to make several commercial products for therapeutic uses. However, in order to get an optimum degradation rate and mechanical properties, these materials could be mixed with polymers. However, the biodegradation rate should match the rate of new bone formation to gradually transfer the active loads to the healing bone and limit the likely unfavorable effects frequently reported with the long-term use of permanent implants. Difficulties such as poor mechanical qualities, uncontrollable BGs degradation, expensive production methods, batch variance, the immunogenicity of natural polymers, lack of biological activity, and hazardous byproducts of composites pose the necessity for further research [36,108,109,110].

On the other hand, strategies based on stem cells have demonstrated the feasibility of approaches that may be transformed into acceptable therapeutic products. However, the research primarily focuses on developing scaffolds with advanced biomaterials, and there aren’t enough studies on cell-biomaterial systems used for tissue reconstruction. Furthermore, precise techniques for harvesting, cultivating, and seeding stem cells on scaffolds to create tissue equivalent to the native tissues in terms of quality and stability are required [216].

There has yet to be a medical device approved by regulatory agencies based on the composite of gelatin and BGs. However, several gelatin-based products exist on the market. Gelatin is regularly used in hydrogels, in inks for bioprinting, and in drug delivery applications [1,2]. Over 20 BG-based medical devices have been approved for clinical use in tissue engineering. Polymer composites have been used in commercial BG devices but have shown a preference towards synthetic polymers for cement-based applications. Individually, these components have been proven successful in the clinic and will soon be used together for a more optimized medical device [36].

To achieve a complete healing process, the more advanced methods may combine the introduction of stem cells, biofactors together with osteoconductive/inductive BGs. The accomplishment of these strategies relies on a deep comprehension of the numerous molecular procedures dealing with the induction of tissue regeneration. This will result in identifying specific BG compositions, biofactors, gelatin, and kinetics required for tissue repair. This article and the next section present some efforts to address these issues, but further research is necessary to fill the lacunas.

## 8. Conclusions and Perspective

This review aimed to provide a more comprehensive and critical explanation of gelatin/BG combinations in the field of tissue engineering. More than 50 relevant publications were discussed from the perspective of processing and physical, mechanical, and biological properties of these composites and/or hybrids, which could show their potential in repairing soft and hard tissues. Table 3 highlights research findings for gelatin/BG composites that have been published in the last five years.

The combination of organic (polymer) and inorganic phases (micro-nano particles of bioactive glass) offers a promising route to capabilities superior to those exhibited by either component alone. For example, it is unanimous in the studies discussed here that gelatin samples modified with bio-glass have better mechanical properties and greater bioactivity, which is highly valued, especially for implants in bone tissue. In addition, all authors who targeted their composites/hybrids for soft tissue regeneration reported greater angiogenesis and faster healing, attested in various wound models.

These results are due to the release of bioactive ions that stimulate biological metabolisms active in the regenerative process. Si, Ca and P ions (present in most BG compositions) contribute to the secretion of growth factors, protein expression, collagen formation and recruitment of cells (such as osteoblasts and fibroblasts) to the lesion site. There is also the possibility of altering bio-glasses’ formulation with metallic ions, which can enhance and/or introduce new properties as required by the application. Chen et al. [27] reported the emergence of antibacterial activity after glass modification with Ce (gelatin/BG-Ce system), while Zheng et al. [133] noticed an increase in the inhibition of these microorganisms after the addition of Cu in the composite based on gelatin and BG, which had already demonstrated an antibacterial effect. Afghah et al. [25], on the other hand, mentioned an anti-inflammatory effect and the acceleration of healing as consequences of the addition of Ag_2_O in the gelatin-PCL/BG system. Dozens of other therapeutic elements can be tested for developing such composites. They can confer multiple properties, from antipathogenic to anticancer. In both cases, the scaffolds/composites can contribute significantly to the healing process and simultaneously combat pathogens and malignancies.

The processing of these materials is considerably versatile in search of the ideal biological performance: other polymers can be used to compose the gelatin matrix (chitosan, alginate, PCL and hyaluronic acid), functionalization agents (such as GPTMS), pyrogenic agents, crosslinkers and different synthesis methods. However, some issues need to be considered when combining different phases, one of them being the concentration of components. In some cases, the addition of bioactive glass can inhibit or suppress the proliferation of cells within the matrix, either by closing the pores of the structure due to the high concentration of particles or by the natural cytotoxicity of metals (when dopants are used in the composition of BG, for example).

Zhou et al. [210] reported less swelling when the percentage of BG increased from 5 to 30 wt% in the gelatin-hyaluronic acid matrix. In situations like this, despite showing acceptable cell viability, the structure with more closed pores directly influences the cell adhesion and proliferation capacity, affecting the material’s performance in vivo. The opposite can also happen: very low concentrations of bio-glass can drive the composite away from the expected ideal behavior, including the delay of bioactive activity [30]. 

Therefore, parameters such as composition and proportion of precursors used in synthesizing these composites must be well selected. As for the cytotoxic effect, a possible way to mitigate it is by adding another dopant to the vitreous structure. Aqib et al. [121] observed that cell viability was higher for the Ag_2_O–SrO association compared to the sample containing only Ag_2_O. Similar results were obtained by Nawaz et al. [218] through the association of Ag and Mn ions. To achieve this, careful structural engineering of glass is necessary, considering the correlation between structure and degradation. Again, there is a plethora of choices, such as investigating the impact of mixed alkali or modifier effect on bioactive glass dissolution.

Another problem is the toxicity of solvents used, e.g., in the promising electrospinning processing route. Kolan et al. [214] discovered reduced cell viability for the electrospun hybrid fibers due to the PLA matrix solvent, which can also occur with chemical crosslinking procedures. This limitation has already been discussed by Chen et al. [219], as well as other aspects that may compromise obtaining organic-inorganic fibers, including particle size and pore conservation. The authors also propose using thermally induced phase separation methods and electrostatic crosslinking as viable alternatives.

Finally, composites and hybrids based on gelatin and BGs are viable current and future proposals for healing tissue defects, with a tendency to expand 3D and 4D printing techniques to mimic tissue-like structures. In the bone segment, there is still a gap between the mechanical strength achieved by these materials and that of cortical bone, although many tests to increase the mechanical stability of composites have been successful. Extending the application of these systems beyond skin lesions would require more in vivo research into their potential in neurological, muscular, and cartilaginous tissues. Another route that can be explored is the transport of drugs in gelatin/BG composites, aiming to intensify the protection against infections and treat other pathologies, such as cancer [139,220]. Here, some authors mentioned the introduction of ciprofloxacin [151] and vancomycin [122,212].

The objectives above can only be achieved through the cooperative efforts of scientists working in the fields of glass, polymers, biology, microbiology, and medicine.

## Figures and Tables

**Table 2:** Therapeutic ions in bioactive glasses and the respective biological properties conferred on the system.

Ion	Properties	References
Ag	Exhibits bactericidal and anti-inflammatory actions Promotes bone formation in vivo Enhances VEGF expression	[23,25,28,106,121,122,123]
B	Stimulates bone formation Increases collagen gene expression Induces angiogenesis	[119,124,125,126]
Ba	Exhibits anti-inflammatory effect Increases cell migration Effective healer of gastric ulcers Radiopacifier	[127,128,129]
Ce	Promotes angiogenesis It has an antibacterial effect Increases collagen production and osteoblastic differentiation	[27,109,119]
Cl	Increases the degradation rate Stimulates the apatite formation	[130,131]
Cu	It has an antibacterial/antimicrobial effect It favors osteogenesis and angiogenesis	[119,132,133]
F	Exhibits antibacterial and anti-inflammatory action Stimulates osteoblast activity when inserted in moderate concentrations Favors the formation of fluorapatite (FAp)	[119,134]
Fe	It is promising for hyperthermia therapy Induces the ferroptosis of tumor cells Stimulates osteoblastic proliferation	[135,136,137]
Ga	Exhibits antibacterial and bacteriostatic action Effective against sarcoma cells Promotes apatite formation	[119,138,139,140]
Li	Induces osteoblastic cell activity Stimulates angiogenesis	[119,141,142,143]
Mg	Stimulates the expression of type I collagen Induces alkaline phosphatase (ALP) activity Increases angiogenesis	[17,144,145,146]
Mn	Increases in vitro bioactivity Promotes greater mechanical strength Exhibits antibacterial effect	[147,148,149]
Sr	Improves mechanical stability Increases in vitro bioactivity Increases bone cell adhesion and stability May optimize the treatment of osteoporosis Radiopacifier	[119,129,150,151,152,153]
Zn	Induces cell proliferation and bone formation It has antibacterial activity Increases mechanical stability It has an anti-inflammatory effect Radiopacifier	[119,129,144,154]
Zr	Produces greater mechanical strength Exhibits antibacterial effect Improves the proliferation of osteoblastic cells Radiopacifier	[116,129,155,156]

**Table 3:** Summary of research results for gelatin/BG composites published in the last 5 years.

Composite	Type	Method	Main Results	Reference
Gelatin/BG 55SiO_2_–24CaO–6P_2_O_5_–15B_2_O_3_	Hydrogels/ Scaffolds	Lyophilization	Notable increase in bioactivity and durability	[20]
Gelatin-chitosan-polyethylene oxide/ Ag-doped BG 45SiO_2_–24.5CaO–24.5Na_2_O–6P_2_O_5_	Nanofibers	Electrospinning	Showed antibacterial activity against gram-positive and gram-negative species; induced a considerable reduction in wound area between 3 and 7 days	[23]
Gelatin- polycaprolactone/ BG 60SiO_2_–30CaO–8P_2_O_5_–2Ag_2_O	Hydrogels/ Scaffolds	Electrospinning	Induced tissue granulation and proliferation of fibroblast cells after the first week of implantation	[25]
Gelatin/BG SiO_2_–CaO–P_2_O_5_–CeO_2_	Hydrogels	Mixing solution	Promoted a wound closing rate of 94.85 ± 2.33% and antibacterial effect against *E. coli* and *S. aureuse*	[27]
Gelatin/BG 64SiO_2_–5P_2_O_5_–26CaO–5MgO	Scaffolds	Freeze-drying	Confirmed antibacterial activity and proliferation of fibroblasts after 3 days	[28]
Gelatin-alginate/BG 45SiO_2_–24.5CaO–24.5Na_2_O–6P_2_O_5_	Scaffolds	Lyophilization	Greater elastic recovery and in vitro degradation rate	[30]
Gelatin-chitosan /BG 50SiO_2_–10P_2_O_5_–34CaO–5SrO–1Ag_2_O	Coatings	Electrophoretic deposition	Increase in the bioactivity and antibacterial action against gram-negative species	[121]
Gelatin/Ag-doped BG 58SiO_2_–33CaO–9P_2_O_5_	Scaffolds	Commercial sponges loaded with BG suspension	Greater filling of the defect with newly formed bone tissue and promising release of vancomycin	[122]
Gelatin-silk fibroin/Cu-doped BG	Hydrogels/ Scaffolds	3D printing	Promotion of significant vascularization and osteogenesis	[132]
Gelatin/BG 95SiO_2_–2.5CaO–2.5CuO	Scaffolds	Foam replica	Increase in the osteogenic activity and the antibacterial effect	[133]
Gelatin/BG SiO_2_–CaO–P_2_O_5_–MgO–ZnO	Scaffolds	Freeze-drying	Promotion of antibacterial activity, stimulation of type I collagen expression and alkaline phosphatase (ALP) activity	[144]
Gelatin/BG 50SiO_2_–35CaO–10P_2_O_5_–5MnO	Scaffolds	Foam replica	Exhibited higher cell viability in vitro and five times higher compressive strength	[147]
Gelatin/Sr-modified BG SiO_2_–CaO–P_2_O_5_	Scaffolds	Freeze-drying	Accelerated degradation rate, improved compressive strength and elastic modulus	[150]
Gelatin/BG 45P_2_O_5_–24CaO–21Na_2_O–5SrO–5Fe_2_O_3_	Scaffolds	Freeze-drying	Sustained release of ciprofloxacin	[151]
Gelatin/BG SiO_2_–CaO–SrO–P_2_O_5_	Scaffolds	Freeze-drying	Promoted bone tissue formation and the presence of mature collagen after 4 weeks	[152]
Gelatin-sodium alginate/BG 80SiO_2_–16CaO–4P_2_O_5_ mol	Scaffolds	3D printing	Exhibited high biocompatibility, biodegradability and osteogenesis	[180]
GelMa/BG 40SiO_2_–45CaO–15P_2_O_5_	Hydrogels/ Scaffolds	Lyophilization	Promotion of cell adhesion, proliferation and osteogenic differentiation	[187]
Gelatin/BG 54.2SiO_2_–35CaO–10.8P_2_O_5_	Scaffolds	Casting and freeze-drying	Good in vitro mineralization and biocompatibility; stiffness of 50–60 kPa, suitable for dental pulp regeneration	[188]
Gelatin-dialdehyde alginate/Cu-doped BG 85SiO_2_–15CaO	Scaffolds	3D printing	Increase in ALP activity and apatite deposition in vitro 3 days	[192]
Gelatin- polycaprolactone/ BG 45SiO_2_–24.5CaO–6P_2_O_5_	Fibers	Electrospinning	Higher hydrophilic, swelling, tensile strength, elastic modulus and ductility; apatite deposition after 14 days	[194]
Gelatin-GPTMS/ BGs 54.6SiO_2_–22.1CaO–7.9K_2_O–7.7MgO–6Na_2_O–1.7P_2_O_5_ and 43.7SiO_2_–22.1CaO–7.9K_2_O–7.7MgO–6Na_2_O–1.7P_2_O_5_–10.9B_2_O_3_	Scaffolds	Freeze-drying	Precipitation of hydroxyapatite after 2 weeks	[195]
GelMa/BG 45SiO_2_–24.5CaO–6P_2_O_5_	Hydrogels	Mixing solution and sonication	Increase in the proliferation of osteoblastic cells, in vitro cell viability, ALP activity and mechanical properties	[196]
Gelatin/BG 64SiO_2_–31CaO–5P_2_O_5_	Scaffolds	Lyophilization and lamination	Considerable cell viability and in vitro bioactivity; tissue growth increased between 4 and 12 weeks	[197]
Gelatin-chitosan/ BG 58SiO_2_–40CaO–5P_2_O_5_	Scaffolds	Lyophilization	Increase of ~80% in the amount of new bone tissue formed after 8 weeks of implantation	[200]
Gelatin/BG 60SiO_2_–36CaO–4P_2_O_5_	Scaffolds	Lyophilization	Significant osseointegration in the initial phase of implantation	[201]
Gelatin/BG 45SiO_2_–24.5CaO–24.5Na_2_O–6P_2_O_5_	Scaffolds	Freeze-drying	Newly formed bone tissue after 6 weeks	[202]
Gelatin- hyaluronic acid/BG 60SiO_2_–36CaO–4P_2_O_5_	Hydrogels	Mixing solution	Effective revascularization and bone regeneration within 8 weeks	[203]
Gelatin-GPTMS/ BG 58SiO_2_–33CaO–9P_2_O_5_ and graphene oxide	Scaffolds	Gas foam	Showed biocompatible and stimulated cell adhesion and proliferation in vitro	[208]
GelMa-collagen/BG	Scaffolds	Lyophilization	Induced biocompatibility, adhesion, proliferation, and cell differentiation, culminating in nerve fibers formation	[211]
Gelatin-alginate/ BG 53B_2_O_3_–20CaO–12K_2_O–6Na_2_O–5MgO–4P_2_O_5_	Scaffolds	3D printing	Improved mechanical properties and cell viability	[213]
Gelatin- alginate/PLA/ BG 53B_2_O_3_–20CaO–12K_2_O–6Na_2_O–5MgO–4P_2_O_5_	Scaffolds	3D printing	Demonstrated faster dissolution and bioactivity in 3D cell culture conditions	[214]
Gelatin-hyaluronic acid/BG 45SiO_2_–24.5CaO–24.5Na_2_O–6P_2_O_5_	Scaffolds	3D printing	Showed bioactivity and greater surface reactivity; increased the modulus of elasticity	[215]
Gelatin-chitosan/BG 64SiO_2_–27CaO–4MgO–5P_2_O_5_ and GPTMS	Injectable pastes	Mixing solution and air drying	Superior mechanical resistance; improved the metabolic activity of cells and supported stem cells’ osteogenic differentiation in a 3D model	[217]

## Data Availability

Not applicable.
